# Genetic characterization of three *qnrS1*-harbouring multidrug-resistance plasmids and *qnrS1*-containing transposons circulating in Ho Chi Minh City, Vietnam

**DOI:** 10.1099/jmm.0.000100

**Published:** 2015-08

**Authors:** Vien Le, Nguyen Thi Khanh Nhu, Ana Cerdeno-Tarraga, James I. Campbell, Ha Thanh Tuyen, Tran Do Hoang Nhu, Pham Thi Thanh Tam, Constance Schultsz, Guy Thwaites, Nicholas R. Thomson, Stephen Baker

**Affiliations:** ^1^​ Hospital for Tropical Diseases, Wellcome Trust Major Overseas Programme, Oxford University Clinical Research Unit, Ho Chi Minh City, Vietnam; ^2^​ Division of Infectious Diseases, Department of Medicine, University of California, San Francisco, CA, USA; ^3^​ School of Chemistry and Molecular Biosciences, University of Queensland, Brisbane, Australia; ^4^​ EMBL-European Bioinformatics Institute, Hinxton, Cambridge, UK; ^5^​ Centre for Tropical Medicine, Nuffield Department of Clinical Medicine, Oxford University, UK; ^6^​ Department of Medical Microbiology, Academic Medical Centre, University of Amsterdam, The Netherlands; ^7^​ Wellcome Trust Sanger Institute, Hinxton, Cambridge, UK; ^8^​ London School of Hygiene and Tropical Medicine, London, UK

## Abstract

Plasmid-mediated quinolone resistance (PMQR) refers to a family of closely related genes that confer decreased susceptibility to fluoroquinolones. PMQR genes are generally associated with integrons and/or plasmids that carry additional antimicrobial resistance genes active against a range of antimicrobials. In Ho Chi Minh City (HCMC), Vietnam, we have previously shown a high frequency of PMQR genes within commensal *Enterobacteriaceae*. However, there are limited available sequence data detailing the genetic context in which the PMQR genes reside, and a lack of understanding of how these genes spread across the *Enterobacteriaceae*. Here, we aimed to determine the genetic background facilitating the spread and maintenance of *qnrS1,* the dominant PMQR gene circulating in HCMC. We sequenced three *qnrS1*-carrying plasmids in their entirety to understand the genetic context of these *qnrS1*-embedded plasmids and also the association of *qnrS1*-mediated quinolone resistance with other antimicrobial resistance phenotypes. Annotation of the three *qnrS1*-containing plasmids revealed a *qnrS1*-containing transposon with a closely related structure. We screened 112 *qnrS1*-positive commensal *Enterobacteriaceae* isolated in the community and in a hospital in HCMC to detect the common transposon structure. We found the same transposon structure to be present in 71.4 % (45/63) of *qnrS1*-positive hospital isolates and in 36.7 % (18/49) of *qnrS1*-positive isolates from the community. The resulting sequence analysis of the *qnrS1* environment suggested that *qnrS1* genes are widely distributed and are mobilized on elements with a common genetic background. Our data add additional insight into mechanisms that facilitate resistance to multiple antimicrobials in Gram-negative bacteria in Vietnam.

## Introduction

Fluoroquinolones are among the current first line of drugs in Vietnam for treating infections ranging from pneumonia to diarrhoea to bacteraemia. Since the mid-1990s, resistance to quinolones has been increasing steadily in multiple organisms causing respiratory tract infections, diarrhoea and bacteraemia (Holt *et al.*, 2013; Nga *et al.*, 2012; Nhu *et al.*, 2014). The emergence of fluoroquinolone resistance in these pathogens and other bacteria represents a clear threat to the effective treatment of common bacterial infections. Resistance to quinolones occurs commonly via mutations in the gene of the target enzyme, DNA gyrase, but can also be plasmid mediated. Plasmid-mediated quinolone resistance (PMQR), in the form of a *qnr* gene, was first described in 1998 in a *Klebsiella pneumoniae* isolate from a patient with urinary tract infection in North America (Martínez-Martínez *et al.*, 1998). Since this first report, multiple studies have described a range of PMQR determinants found within the *Enterobacteriaceae* and other bacterial families (Strahilevitz *et al.*, 2009). To date, five *qnr* genes have been described and are distinguished on the basis of their sequence homology: *qnrA*, *qnrB* and *qnrS1*, and more recently *qnrC* and *qnrD* (Cavaco *et al.*, 2009; Hata *et al.*, 2005; Jacoby *et al.*, 2006; Martínez-Martínez *et al.*, 1998; Wang *et al.*, 2009). The *qnr* genes appear to be highly promiscuous, having the capacity to become rapidly disseminated among related and unrelated hosts. The transmissibility of the *qnr* genes makes the genomic mechanisms facilitating their movement of considerable interest and of relevance in the community and in healthcare settings. Of the five *qnr* genes identified, only the genetic contexts of *qnrA* and *qnrB* have been comprehensively described. These genes are commonly located within complex *sul1*-type class 1 integrons (Garnier *et al.*, 2006; Poirel *et al.*, 2006). The context of *qnrS* is less well described, but there are reports of the gene being located within a gene cluster flanked by IS*26* transposases (Chen *et al.*, 2006; Hu *et al.*, 2008). However, it is currently unknown if the *qnrS* gene is always associated with IS*26* or if *qnrS* can be transferred and/or maintained by other, unrelated, mobile elements.

Several *qnrS1-*carrying plasmids have been described in the literature and have publicly available nucleotide sequences. These plasmids range in size and belong to various incompatibility groups including IncN (Dobiasova *et al.*, 2013; Literak *et al.*, 2010), IncI1 (Dobiasova *et al.*, 2013), IncX1 (Dobiasova *et al.*, 2013; Literak *et al.*, 2010) and IncX2 (Literak *et al.*, 2010; Sumrall *et al.*, 2014). These *qnrS1*-encoding plasmids have been identified in Asia and Europe and in a range of Gram-negative bacteria including *K. pneumoniae*, *Escherichia coli* and *Enterobacter aerogenes* (Dobiasova *et al.*, 2013; Hu *et al.*, 2008; Park *et al.*, 2009; Sumrall *et al.*, 2014). Two of the earliest and best-described *qnrS1*-encoding plasmids are pTPqnrS-1a and pK245. Plasmid pTPqnrS-1a is a 10 kb replicon, and was isolated from a multidrug-resistant (MDR) *Salmonella* Typhimurium DT193 in the UK (Kehrenberg *et al.*, 2007). The second, pK245, was characterized in a clinical isolate of *K. pneumoniae* originating in Taiwan (Chen *et al.*, 2006). In contrast to pTPqnrS-1a, pK245 is a large MDR plasmid of approximately 100 kb. The MDR phenotype of pK245 was demonstrated by transferring this plasmid into an antimicrobial-susceptible *Escherichia coli* strain by electrotransformation (Chen *et al.*, 2006). The pK245-positive transformant showed an increase in MICs to multiple classes of antimicrobials, including aminoglycosides, β-lactams and (fluoro)quinolones and had an extended-spectrum β-lactamase (ESBL) phenotype (Chen *et al.*, 2006). Comparative sequence analysis of available *qnrS1* plasmids revealed that the genetic architecture surrounding the *qnrS1* gene is identical between pTPqnrS-1a and pK245, and they additionally sharing a high sequence identity with the *qnrS1* genetic region in other partial plasmid sequences, including pAH0376 from *Shigella* *flexneri* (Hata *et al.*, 2005) and pINF5 from *Salmonella* Infantis (Kehrenberg *et al.*, 2006).

In a study investigating the distribution of PMQR determinants in *Enterobacteriaceae* isolated from hospitalized patients and healthy volunteers from Ho Chi Minh City (HCMC), Vietnam, we found an exceptionally high prevalence of the *qnrS1* genes in both hospital (63/139, 45 %) and community (49/413, 12 %) bacterial isolates (Vien *et al.*, 2009). We therefore hypothesized that *qnrS1* was embedded on a highly mobile and conserved genetic element, which was contributing to the spread and the apparent success of *qnrS1* across the *Enterobacteriaceae* in this setting. In this current study, we aimed to characterize the dominant *qnrS1*-containing elements circulating in *Enterobacteriaceae* isolated from hospital patients and community volunteers in HCMC to understand if *qnrS1* is being disseminated on one or more elements by defining their genetic context. To achieve this, we selected three *qnrS1*-containing plasmids, broadly representative of those found to be circulating in the hospital and community environments (Vien *et al.*, 2012), for DNA sequencing and analysis in their entirety. The resulting sequence analysis of the *qnrS1*-containing mobile elements has broadened our knowledge of the genetic architecture surrounding the *qnrS1* gene and added insight into MDR mechanisms that are circulating within these differing bacterial environments in Vietnam.

## Methods

### Bacterial strains

A total of 115 *qnrS1*-positive *Enterobacteriaceae* strains (38 *Escherichia coli*, 69 *K. pneumoniae* and eight from other *Enterobacteriaceae* specie*s*) were selected for analysis in this study. All of the strains have been described previously and were isolated from patients admitted to the tetanus ward of the Hospital for Tropical Diseases (HTD) in HCMC, Vietnam, between May and October 2004 and between June and November 2005 or from healthy volunteers participating in a typhoid vaccine study in 2005 and 2006 (Tran *et al.*, 2010). The presence of the *qnrS1* gene in these strains has been previously confirmed and described (Vien *et al.*, 2009).

Three *qnrS1*-containing plasmids identified previously (Vien *et al.*, 2012) were selected to be broadly representative of *qnrS1*-containing plasmids circulating in our setting (i.e. harbouring strain, hospital/community infections and size) and were sequenced in their entirety, assembled and annotated gene by gene in comparison to sequences available in public databases. These plasmids (selected out of the 115 *qnrS1*-positive *Enterobacteriaceae* strains) were pE66An (*Escherichia coli* host), pK18An (*K. pneumoniae* host) and pK1HV (*K. pneumoniae* host). A summary of the basic features of these plasmids is given in Table 1.

**Table 1:** Features of the three sequenced *qnrS1*-containing plasmids

Characteristic	pE66An	pK18An	pK1HV
Original strain	*Escherichia coli*	*K. pneumoniae*	*K. pneumoniae*
Source	Hospital	Hospital	Community
Size (kb)	80.105	51.160	133.191
G+C (mol%)	51.25	51.32	52.5
Inc group	IncN	IncN	IncFII
Predicted coding sequences	109	72	167
Essential function genes	22	15	14
Conjugative system genes	16	6	21
Resistance genes	7	5	11
IS elements	IS*26*, IS*6100*, IS*6100*903D, IS*Ecp1*	IS*26*, IS*10*, IS*5*	IS*26*, IS*4*, IS*1414*
Integron	Integron 1	Integron 1	Integron 1

### Plasmid sequencing and annotation

The three *qnrS1*-containing plasmids were sequenced at the Wellcome Trust Sanger Institute in the UK using conventional Sanger sequencing methods as described previously (Parkhill *et al.*, 2001). The plasmid sequences were annotated and analysed using Artemis (Rutherford *et al.*, 2000) and aligned and compared using the Artemis Comparison Tool (Carver *et al.*, 2008). Plasmid circularization and graphical representations were performed using DNA Plotter software (Carver *et al.*, 2009).

### PCR amplification for the qnrS1-containing transposon and RFLP typing

Total genomic DNA from 112 *qnrS1* PCR amplification-positive *Enterobacteriaceae* isolates was extracted using a Wizard Genomic DNA purification kit (Promega), according to the manufacturer's specifications. PCR amplification for the *qnrS1*-containing transposon from the extracted genomic DNA was performed under the following condition: 94 °C for 10 s, 55 °C for 30 s and 68 °C for 6 min for 35 cycles. Amplification was performed using the Expand Long Template PCR System (Roche) using the primers Trans-qnrS-F (5′-CAGGAAGAGGCATTGTCAAAGG-3′) and Trans-qnrS-R (5′-GGTGCTTGTCAGCGTAAA-3′). These primers were designed using Primer Express 5 software (Applied Biosystems, Life Technologies) and their specificity was assessed *in silico* using blastn (http://blast.ncbi.nlm.nih.gov/Blast.cgi). The resulting PCR amplicons were examined by electrophoresis and UV visualization on 2 % agarose gels containing 2 % ethidium bromide. The PCR amplicons containing the *qnrS1* gene were typed using RFLP with three different enzymes: *Eco*RV, *Hin*dIII and *Pvu*II (New England Biolabs). The restriction-digested PCR amplicons were analysed by gel electrophoresis for 2 h on a 1 % agarose gel, stained with 2 % ethidium bromide and examined under UV light. The restriction fragments were sized and compared for group typing using Bionumerics software (Applied Maths).

### Primer-walking sequencing

The *qnrS1*-containing transposon from the K34N strain was sequenced using primer walking. The sequencing reaction was performed in a 20 μl reaction containing 4 μl Big Dye Terminator, 2 μl buffer, 20 ng genomic DNA and distilled water up to 20 μl. Each fragment was repeated four times using an ABI 3130XL machine (Applied Biosystems, Life Technologies). All sequences were assembled using Vector NTI software (Life Technologies).

### Electrotransformation

PCR-negative isolates for the *qnrS1*-containing transposon were analysed for the presence of subfamilies of the known transposon. Plasmid DNA from these negative isolates was extracted using a Qiagen Midi Prep Plasmid DNA Extraction kit, as per the manufacturer's recommendations. *Escherichia coli* TOP10 cells (Invitrogen, Life Technologies) were transformed with isolated plasmid DNA by a Bio-Rad gene pulser, using conditions recommended by the manufacturer (Invitrogen, Life Technologies). Transformants were selected on Luria–Bertani medium supplemented with 0.03 mg ciprofloxacin l^− 1^. Plasmid DNA from these transformants was extracted by the method of Kado & Liu (1981), examined on an agarose gel for the presence of only one plasmid and then subjected to PCR amplification for the *qnrS1* gene to ensure transformation of the appropriate plasmid.

### Southern blot analysis

Isolates containing a subfamily of known *qnrS1*-containing transposons were detected by Southern blotting with two different probes: qnrS1 and bla_LAP-2_, using the primers described for amplification of the qnrS1 region. Plasmid DNA from the transformants was extracted using a Qiagen Midi Prep Plasmid DNA Extraction kit, as per manufacturer's recommendations. These plasmids were then digested with *Eco*RI and duplicates were run on a gel. The gel was subsequently transferred to a membrane. The membrane was cut into two pieces and each was hybridized with one of the probes, qnrS1 or bla_LAP-2_. If an isolate had signal with both probes binding to the same fragment, it was assigned as carrying a subfamily of a *qnrS1*-containing transposons.

## Results and Discussion

### Global comparison of the three *qnrS1*-containing plasmids

**Fig. 1:**
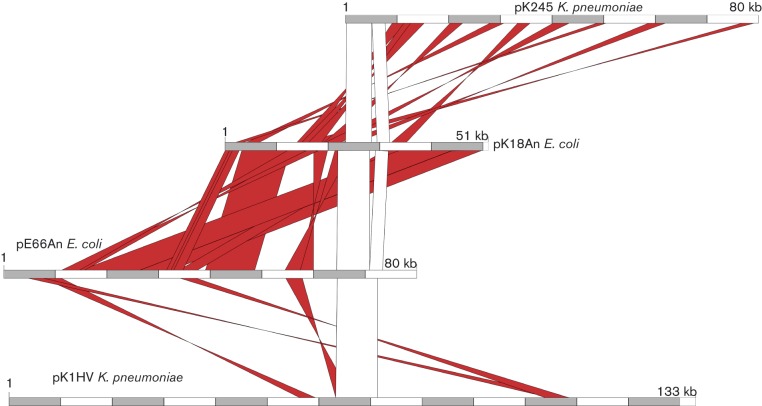
Linear DNA sequence alignments of pE66An, pK18An and pK1HV against plasmid pK245 centred at the *qnrS1*-encoding region. Regions of DNA identity of 99 % or greater are linked by red blocks. The open box is the region containing the *qnrS1*-harbouring transposon, which is identical in all four plasmids except for a 980 bp insert in pK18An. The sizes of the plasmids are shown, with each shaded/open block representing 10 kbp.

Figure 1 shows a global DNA alignment of the three sequenced plasmids and a previously sequenced *qnrS1*-containing plasmid (pK245) identified in a *K. pneumoniae* isolate from Taiwan as a comparator (Chen *et al.*, 2006). These alignments showed that the two plasmids isolated independently from different bacterial genera within the hospital environment (pE66An and pK18An) exhibited substantial gene synteny with each other, but generally shared a lower degree of DNA homology with the plasmid identified in a community isolate (pK1HV).

### Plasmid pE66An

**Fig. 2:**
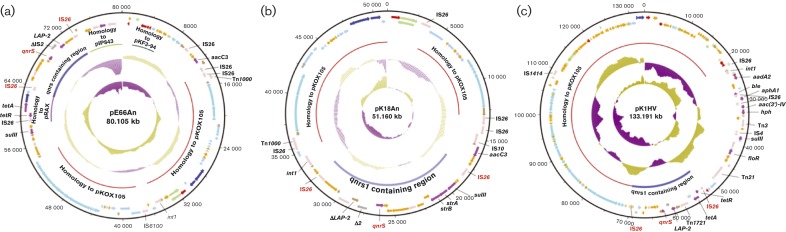
Annotated circular plasmid maps of pE66An, pK18An and pK1HV orientated from the origin of replication. (a) pE66An; (b) pK18An; (c) pK1HV. Identified and annotated ORFs are colour coded on each of the three plasmids: red, plasmid replication; dark blue, inorganic/metal/UV resistance; sky blue, conjugal transfer; dark pink, antimicrobial resistance; light green, unknown; light blue, regulators; orange, conserved hypothetical; brown, pseudogenes or partial genes; light pink, IS elements. The arrow annotation shows the strand on which the ORF is located. The inner circle shows the GC skew ([GC]/[G+C]) and the next outer circle shows G+C (mol%) plot. Fragments with substantial DNA homology to other sequenced plasmids and the *qnrS*-encoding region are highlighted.

The *qnrS1*-encoding plasmid pE66An was extracted from an *Escherichia coli* strain isolated from a rectal swab taken from a patient admitted to the tetanus ward of the HTD in HCMC. Plasmid pE66An is 80 105 bp with an approximately neutral G+C content of 51.52 mol% (Fig. 2). After complete annotation of the plasmid sequence, 109 predicted coding sequences (CDSs) were identified; the protein products of seven of these CDSs were predicted to be associated with resistance to a variety of antimicrobial classes. These antimicrobial resistance genes were: *aacC3* (gentamicin), *sulII* (sulfonamides), *tetR* and *tetA* (tetracyclines), *qnrS1* (quinolones), *bla*_LAP-2_ (β-lactams) and *bla*_CTX-M-14_ (third-generation cephalosporins). An association between the *qnrA* gene and ESBL-encoding genes has been reported previously (Castanheira *et al.*, 2007; Hamouda *et al.*, 2008; Lavigne *et al.*, 2006). The *bla*_CTX-M-14_ gene within pE66An was adjacent to the element IS*Ecp1*. The insertion element IS*Ecp1* has been shown previously to mediate the transfer of *bla*_CTX-M-14_ (Bou *et al.*, 2002). As the *bla*_CTX-M-14_ gene is in association with this IS*Ecp1* insertion element, the potential for dissemination of this gene to other plasmids or transposable elements is likely to be enhanced.

Comparative analysis showed that two regions within plasmid pE66An, a 16.7 and a 10.2 kb region, exhibited significant DNA sequence similarity to regions within the previously described *Klebsiella oxytoca* plasmid pKOX105 (Carattoli *et al.*, 2010) and three smaller regions (5.9, 5.2 and 3.5 kb) within plasmids pIP843 (Cao *et al.*, 2002), pRAx (Fricke *et al.*, 2009) and pKF3-94 (Zhao *et al.*, 2010) from HCMC, Madagascar and China, respectively (Fig. 2a). Plasmid pKOX105 was isolated from a *K. oxytoca* isolate present in the intestinal microbiota of an individual in a long-term care facility in Bolzano, Italy, in 2005. Both pE66An and pKOX105 are IncN plasmids, with each containing the highly conserved IncN plasmid backbone (Carattoli *et al.*, 2010). Plasmid pE66An contains two regions of 10 280 and 5183 bp that encode sequences predicted to be responsible for the conjugal transfer. Indeed, our previous work has shown that pE66An has the capacity to be efficiently conjugated at high frequency into a suitable recipient strain. We therefore concluded that this conjugation system is functionally active (Vien *et al.*, 2009).

In addition to antimicrobial resistance and conjugal transfer functions, plasmid pE66An also encodes genes that suggest that it may be able to adapt to a variety of hosts and environments. For example, we identified a CDS with 99.5 % identity to *frmA*, a class III alcohol dehydrogenase identified in a *Pasteurella piscicida* isolate (Kim & Aoki, 1994), and 98.1 % identity to a class III alcohol dehydrogenase identified in an *Escherichia coli* isolate (Hochhut *et al.*, 2006). The function of the protein product encoded by *frmA* is involved in resistance to formaldehyde and other aldehydes. We predict that *frmA* provides a selective advantage for bacterial hosts in hospital environments where disinfectants containing aldehydes are used (Chen *et al.*, 2006).

### Plasmid pK18An

Plasmid pK18An was carried by a *K. pneumoniae* isolate taken from a rectal swab of a tetanus patient in the HTD in HCMC. The sequence of plasmid pK18An showed it was a circular replicon of 51 160 bp, with a G+C content of 51.32 mol% (Fig. 2). The annotation of pK18An identified 72 CDSs, the functions of six of which were predicted to be associated with resistance to antimicrobials, including *aacC3* (gentamicin), *sulII* (sulfonamides), *strA* and *strB* (streptomycins), *dhfrV* (trimethoprim) and *qnrS1* (quinolones). Plasmid pK18An was also found to harbour a *bla*_LAP-2_ gene in close proximity to *qnrS1*, but that carried an IS*5* element insertion and so was likely to have been inactivated. The *sulII*–*strA*–*strB* genes were located in close proximity to each other and adjacent to an IS*26* element. The *strA*–*strB* genes are often linked with the *sulII* sulfonamide-resistance gene, commonly encoded on broad-host-range non-conjugative plasmids in a range of Gram-negative bacteria found in humans and animals. The usage of streptomycin in clinical and animal medicine has diminished dramatically over the last 10–20 years, yet the persistence of *sulII*–*strA*–*strB* implies that factors other than a direct selection pressure from the antimicrobial are important for the maintenance of these genes (Sundin & Bender, 1996).

Like pE66An, plasmid pK18An shared two large regions, of 10.2 and 9.4 kb, with extensive DNA homology to plasmid pKOX105 (Carattoli *et al.*, 2010) (Fig. 2b). Similarly, plasmid pK18An also contained two regions containing CDSs that are predicted to be responsible for the conjugal transfer, but these operons were disrupted by numerous IS*26* elements. These sequence data probably explain why it was not possible to conjugate pK18An into a recipient *Escherichia coli* under laboratory conditions (Vien *et al.*, 2009). Whilst pK18An was not conjugative, the plasmid sequence was littered with multiple IS elements, particularly surrounding antimicrobial resistance genes, suggesting that such elements may facilitate the independent transfer of these genes to other plasmids.

Plasmids pK18An and pE66An both contained restriction modification systems; pK18An contained an *ecoRIIM* gene and pE66An contained the *ecoRIIR* and *ecoRIIM* genes. The *ecoRIIR* and *ecoRIIM* genes share 98.8 % nucleotide identity with the e*coRII* endonuclease gene (Bhagwat *et al.*, 1990), and have 100 % nucleotide identity with the *Escherichia coli* modification methylase gene, *ecoRII* (Som *et al.*, 1987). In addition to assisting with defence against bacteriophage infection, this restriction modification system has also been reported to contribute to the spread and maintenance of plasmids encoding these systems (Kobayashi, 2001).

### Plasmid pK1HV

Plasmid pK1HV was isolated from a *K. pneumoniae* strain cultured from a healthy child, resident in HCMC. Plasmid pK1HV was the largest of the three sequenced plasmids at 133 191 bp with a G+C content of 52.5 mol% (Fig. 2). Plasmid pK1HV contained 167 predicted CDSs, of which the overwhelming majority were of unknown function. However, pK1HV was also found to carry 11 genes that are associated with resistance to various classes of antimicrobials, including *aadA2* (streptomycin), *ble* (bleomycin), *aphA1* (gentamicin), *aac(3′)-IV* (gentamicin), *hph* (hygromycin), *sulII* (sulfonamides), *forR* (chloramphenicol), *tetR* and *tetA* (tetracyclines), *bla*_LAP-2_ (β-lactams) and *qnrS1* (quinolones). Similar to pE66An, pK1HV also carried an IS*26*–*tetR*–*tetA* complex, which is a common mechanism facilitating the transfer of tetracycline resistance. Plasmid pK1HV also harboured a type 1 integron containing the *dfrA12*–*orfF*–*aadA2* cassette, an antimicrobial resistance region that remains common in contemporarily isolated MDR Gram-negative organisms (Gestal *et al.*, 2005). The presence this *dfrA12*–*orf*–*aadA2*-containing type 1 integron in pK1HV (isolated from the community) again poses questions regarding the ongoing selection of genes encoding resistance to streptomycin.

Comparative sequence analysis of pK1HV with pKOX105 revealed a large contiguous fragment of ∼84 kb shared between both plasmids (Carattoli *et al.*, 2010) (Fig. 2c). All of the predicted genes on this extended fragment of plasmid DNA were mostly conserved but functionally unknown, or were genes proposed to encode components required for conjugal transfer. The remaining regions of pK1HV carried the identified antimicrobial resistance genes and an array of IS elements (Fig. 2c). Whilst pK1HV did not contain the *mucAB* operon, like pE66An and pK18An, it did contain the *imp* operon, which had 85 % nucleotide identity to *impA* and *impB* on the IncI1 plasmid TP110 from a *Salmonella* Typhimurium strain isolated in the UK in 1968 (Lodwick *et al.*, 1990).

### Characterization of *qnrS1*-containing transposons

**Fig. 3:**
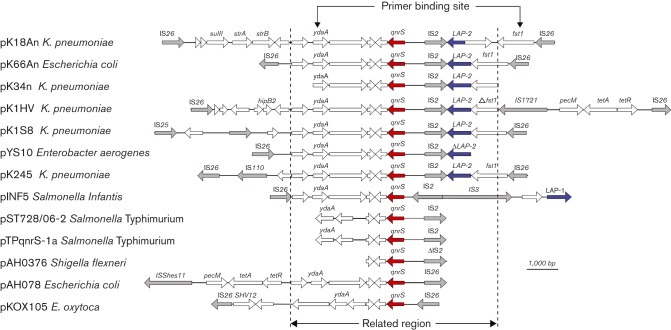
A schematic representation of sequenced *qnrS1*-containing transposons. Graphical representation of the synteny between the *qnrS*-containing transposons between the three plasmids sequenced here (pK18An, pE66An and pK1HV) and other sequenced fragments containing the *qnrS*-encoding region. The plasmids and the host organism in which they were first identified are given. The region with the greatest DNA homology is identified and includes the highlighted ORFs for *qnrS* (red), a putative IS*2* element (grey), a gene encoding a putative resolvase protein (*ydaA*) and three other ORFs encoding hypothetical proteins of unknown function. Additional genes are colour coded: blue, *bla*_LAP-2_; grey, IS elements; white, ORFs without a name encoding hypothetical uncharacterized proteins. The locations of the binding sites for PCR amplification of the transposon are highlighted.

Annotation of the three sequenced *qnrS1*-containing transposons extracted from the plasmid sequences of pE66An, pK18An and pK1HV and their alignments with other described *qnrS1*-containing fragments are shown in Fig. 3. The alignment of *qnrS1*-containing fragments from pE66An, pK18An and pK1HV showed that they were identical, except for pK18An, which contained a 980 bp insertion. The *qnrS1* gene in all of the three plasmids was located within a transposon structure composed of two identical IS*26* elements at either terminal portion of the transposon. In addition to containing the *qnrS1* gene, these transposons also carried the *bla*_LAP-2_ gene, which confers resistance to narrow-spectrum β-lactams. Additionally, this transposon shared a common backbone with other available *qnrS* sequences and contained an IS*2* element, a putative resolvase (*ydaA*) predicted to belong to a family of stress proteins (Beliaev *et al.*, 2002), and three other proteins of unknown function (Park *et al.*, 2009; Wu *et al.*, 2008) (Fig. 3). The G+C content of the *qnrS*-containing transposons was ∼50 mol%, which was slightly lower than the mean G+C content of the sequenced plasmids in their entirety, consistent with the notion that the *qnrS1*-encoding transposons have been inserted into these plasmids via horizontal gene transfer.

### Screening for *qnrSI* transposons in commensal *Enterobacteriaceae* isolated in HCMC

Using the newly generated DNA sequences of the *qnrS1*-containing transposons from the three sequenced plasmids (Fig. 3), we designed PCR primers to amplify and compare *qnrS1-*containing transposons from DNA extracted from 112 *qnrS1-*positive hospital- and community-acquired *Enterobacteriaceae* that have been described previously (Vien *et al.*, 2011, 2012; see Methods). The locations of the primer-binding sites are shown in Fig. 3 and the predicted sizes of PCR amplicons were 6859 bp (pE66An *qnrS1* transposon, type A) and 8059 bp (pK18An *qnrS1* transposon, type B). Seventy-one of the 112 isolates (63.4 %) were PCR amplicon-positive for the *qnrS1*-containing transposon and were of sizes consistent with those described in pE66An and pK18An. The 71 PCR amplicons with known *qnrS1*-containing transposons were subjected to RFLP analysis with *Eco*RV, *Hin*dIII and *Pvu*II. In addition to the two described *qnrS1*-containing transposons that were identified in the three sequenced plasmids, the RFLP mapping patterns from these 71 amplicons also revealed a transposon with a third structure (K34N strain, type C). Using a primer-walking sequencing method, we found that the *qnrS1*-containing transposon in K34N strain was 6652 bp and identical to the *qnrS1*-containing transposon from E66An strain, except for a 200 bp deletion in a gene of unknown function (Fig. 3). We were therefore able to distinguish three related yet distinct *qnrS1*-containing transposons of 6859, 8059 and 6652 bp, which we arbitrarily named types A, B and C, respectively.

**Fig. 4:**
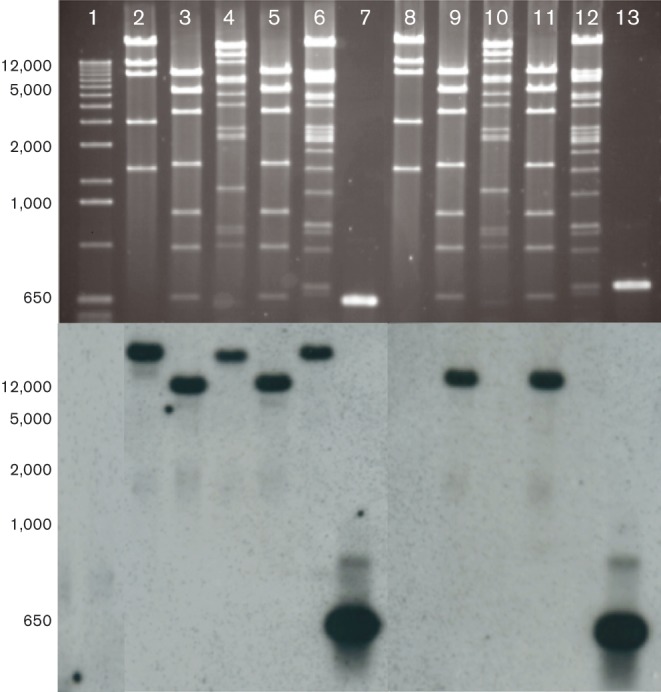
*Eco*RI digestion of *qnrS*-encoding plasmids hybridized with the *qnrS1* and *bla*_*LAP-2*_ probes. Upper panel: agarose gel electrophoresis of *qnrS*-encoding plasmids after digestion with *Eco*RI. The resulting patterns are duplicate digestions from five plasmids after conjugation. Lanes: 2 and 8, isolate LTMV18; 3 and 9, isolate LTMV33; 4 and 10, isolate LTMV6; 5 and 11, isolate LTMV30; 6 and 12, isolate LTMV1. The ladder (lane 1) is 1 kb Plus (Invitrogen) with sizes shown in kb. Lanes 7 and 13 contain the PCR amplicons of *qnrS* and *bla*_LAP-2_ as positive controls. Bottom panel (left): hybridization against the *qnrS1* probe after Southern blotting. All five plasmids contained fragments corresponding to probe *qnrS1*. Bottom panel (right): hybridization against the *bla*_LAP-2_ probe after Southern blotting.

The 41 isolates with an undetermined *qnrS1*-containing mobile element were further investigated for the presence of a subfamily of the known transposon. There have been multiple reports regarding the association between *qnrS1* and *bla*_LAP-2_ genes (Cano *et al.*, 2009; Dahmen *et al.*, 2010; Huang *et al.*, 2008; Park *et al.*, 2009; Poirel *et al.*, 2006, 2007). Yet, due to a lack of a PCR amplicon, we hypothesized that some isolates contain both the *qnrS1* and the *bla*_LAP-2_ sequence, but the adjacent regions demonstrate a different structure. A laboratory *Escherichia coli* strain was successfully transformed with a *qnrS1*-encoding plasmid extracted from 27 of the 41 isolates with an undefined *qnrS1*-containing transposon structure. Plasmid DNA from these 27 transformants was extracted and digested with *Eco*RI and individually probed by Southern blotting targeting the *qnrS1* and *bla*_LAP-2_ genes. With the resulting plasmid hybridization of the plasmid DNA extracted from the 27 isolates, only two produced a detectable signal with both *qnrS1* and *bla*_LAP-2_ probes on the same digestion fragment, implying that these two plasmids (and their corresponding hosts) also carry a *qnrS1*-containing transposon of the same subfamily as described in the sequenced plasmids (Fig. 4).

We stratified the proportion of the three *qnrS1*-containing transposons across all the hospital- and community-acquired isolates that were compared (Table 2). Forty-five of the 63 hospital isolates (71.4 %) harboured a *qnrS1*-containing transposon that was identical to pE66An (type A), one isolate (1.6 %) carried a *qnrS1*-containing transposon identical to pK18An (type B) and three isolates (4.8 %) carried a *qnrS1*-containing transposon identical to K34N (type C). Similarly, in the 49 community isolates, there were 18 (36.7 %), one (2 %) and three (6.1 %) isolates carrying type A, B and C *qnrS1*-containing transposons, respectively. However, 14 isolates (22.2 %) from the hospital and 27 isolates (55.1 %) from the community *qnrS1* gene-positive isolates were negative by PCR targeting the *qnrS1*-containing transposon, suggesting that the *qnrS1* gene in these strains is embedded on a different genetic element, which was undeterminable by the described PCR amplification methods used here.

**Table 2:** Distribution of the various *qnrS1*-containing transposons in 122 *Enterobacteriaceae* isolated in HCMC

Strain	Type A	Type B	Type C	Subtype	Unknown
Hospital strains (*n* = 63)					
*Escherichia coli* (*n* = 5)	3 (60 %)	0	0	0	2 (40 %)
*K. pneumoniae* (*n* = 52)	40 (77 %)	1 (2 %)	3 (5.7 %)	0	8 (15.3 %)
Others (*n* = 6)	2 (33.3 %)	0	0	0	4 (66.7 %)
Community strains (*n* = 49)					
*Escherichia coli* (*n* = 32)	13 (40.6 %)	0	0	1 (3.1 %)	18 (56.3 %)
*K. pneumoniae* (*n* = 15)	5 (33.3 %)	1 (6.7 %)	3 (20 %)	1 (6.7 %)	5 (33.3 %)
Others (*n* = 2)	0	0	0	0	2 (100 %)

## Conclusions

Hospital-acquired infections with antimicrobial-resistant organisms can be dangerous, and we have recently shown the problems that can be associated with highly virulent clones of *K. pneumoniae* (Chung The *et al.*, 2015). Here, we have shown that there is a dominant *qnrS1*-containing transposon circulating in *qnrS1*-positive *Enterobacteriaceae* in HCMC, Vietnam, which we found to be present in 71.4 % of *qnrS1*-positive isolates from a hospital and in 36.7 % of *qnrS1*-positive isolates from the community. Moreover, we determined the complete nucleotide sequences of three *qnrS1*-encoding plasmids. These sequences permitted a description of the circulating genes contributing to an MDR phenotype in three bacterial isolates from a hospital and the community, and also provided insights into the means of adaptation of these plasmids within a variety of hosts and environments. Notably, the DNA sequences of the two plasmids isolated from two different bacterial genes in a hospital setting exhibited substantial homology, thus presenting evidence of genetic transfer among nosocomial commensal bacteria. Finally, the annotation of plasmid pE66An provides further evidence for the association of the PMQR gene *qnrS* and the ESBL gene *bla*_CTX-M-14_.

## References

[jmm000100-Beliaev1] Beliaev A.S. Thompson D.K. Fields M.W. Wu L. Lies D.P. Nealson K.H. Zhou J. (2002). Microarray transcription profiling of a *Shewanella oneidensis* *etrA* mutant J Bacteriol 184 4612–4616 10.1128/JB.184.16.4612-4616.2002 . 12142431 PMC135233

[jmm000100-Bhagwat1] Bhagwat A.S. Johnson B. Weule K. Roberts R.J. (1990). Primary sequence of the *Eco*RII endonuclease and properties of its fusions with β-galactosidase J Biol Chem 265 767–773 . 2104830

[jmm000100-Bou1] Bou G. Cartelle M. Tomas M. Canle D. Molina F. Moure R. Eiros J.M. Guerrero A. (2002). Identification and broad dissemination of the CTX-M-14 β-lactamase in different *Escherichia coli* strains in the northwest area of Spain J Clin Microbiol 40 4030–4036 10.1128/JCM.40.11.4030-4036.2002 . 12409370 PMC139670

[jmm000100-Cano1] Cano M.E. Rodríguez-Martínez J.M. Agüero J. Pascual A. Calvo J. García-Lobo J.M. Velasco C. Francia M.V. Martínez-Martínez L. (2009). Detection of plasmid-mediated quinolone resistance genes in clinical isolates of *Enterobacter* spp. in Spain J Clin Microbiol 47 2033–2039 10.1128/JCM.02229-08 . 19386836 PMC2708531

[jmm000100-Cao1] Cao V. Lambert T. Courvalin P. (2002). ColE1-like plasmid pIP843 of *Klebsiella* *pneumoniae* encoding extended-spectrum β-lactamase CTX-M-17 Antimicrob Agents Chemother 46 1212–1217 10.1128/AAC.46.5.1212-1217.2002 . 11959547 PMC127148

[jmm000100-Carattoli1] Carattoli A. Aschbacher R. March A. Larcher C. Livermore D.M. Woodford N. (2010). Complete nucleotide sequence of the IncN plasmid pKOX105 encoding VIM-1, QnrS1 and SHV-12 proteins in Enterobacteriaceae from Bolzano, Italy compared with IncN plasmids encoding KPC enzymes in the USA J Antimicrob Chemother 65 2070–2075 10.1093/jac/dkq269 . 20656680

[jmm000100-Carver1] Carver T. Berriman M. Tivey A. Patel C. Böhme U. Barrell B.G. Parkhill J. Rajandream M.-A. (2008). Artemis and ACT: viewing, annotating and comparing sequences stored in a relational database Bioinformatics 24 2672–2676 10.1093/bioinformatics/btn529 . 18845581 PMC2606163

[jmm000100-Carver12] Carver T. Thomson N. Bleasby A. Berriman M. Parkhill J. (2009). DNAPlotter: circular and linear interactive genome visualization Bioinformatics 25 119–120 10.1093/bioinformatics/btn578 . 18990721 PMC2612626

[jmm000100-Castanheira1] Castanheira M. Pereira A.S. Nicoletti A.G. Pignatari A.C.C. Barth A.L. Gales A.C. (2007). First report of plasmid-mediated *qnrA1* in a ciprofloxacin-resistant *Escherichia coli* strain in Latin America Antimicrob Agents Chemother 51 1527–1529 10.1128/AAC.00780-06 . 17220425 PMC1855464

[jmm000100-Cavaco1] Cavaco L.M. Hasman H. Xia S. Aarestrup F.M. (2009). *qnrD*, a novel gene conferring transferable quinolone resistance in *Salmonella enterica* serovar Kentucky and *Bovis morbificans* strains of human origin Antimicrob Agents Chemother 53 603–608 10.1128/AAC.00997-08 . 19029321 PMC2630628

[jmm000100-Chen1] Chen Y.-T. Shu H.-Y. Li L.-H. Liao T.-L. Wu K.-M. Shiau Y.-R. Yan J.-J. Su I.-J. Tsai S.-F. Lauderdale T.-L. (2006). Complete nucleotide sequence of pK245, a 98-kilobase plasmid conferring quinolone resistance and extended-spectrum-β-lactamase activity in a clinical *Klebsiella pneumoniae* isolate Antimicrob Agents Chemother 50 3861–3866 10.1128/AAC.00456-06 . 16940067 PMC1635178

[jmm000100-Chung1] Chung The H. Karkey A. Pham Thanh D. Boinett C.J. Cain A.K. Ellington M. Baker K.S. Dongol S. Thompson C. other authors (2015). A high-resolution genomic analysis of multidrug-resistant hospital outbreaks of *Klebsiella* *pneumoniae* EMBO Mol Med 7 227–239 10.15252/emmm.201404767 . 25712531 PMC4364942

[jmm000100-Dahmen1] Dahmen S. Poirel L. Mansour W. Bouallègue O. Nordmann P. (2010). Prevalence of plasmid-mediated quinolone resistance determinants in Enterobacteriaceae from Tunisia Clin Microbiol Infect 16 1019–1023 10.1111/j.1469-0691.2009.03010.x . 19650824

[jmm000100-Dobiasova1] Dobiasova H. Dolejska M. Jamborova I. Brhelova E. Blazkova L. Papousek I. Kozlova M. Klimes J. Cizek A. Literak I. (2013). Extended spectrum β-lactamase and fluoroquinolone resistance genes and plasmids among *Escherichia coli* isolates from zoo animals, Czech Republic FEMS Microbiol Ecol 85 604–611 10.1111/1574-6941.12149 . 23679004

[jmm000100-Fricke1] Fricke W.F. Welch T.J. McDermott P.F. Mammel M.K. LeClerc J.E. White D.G. Cebula T.A. Ravel J. (2009). Comparative genomics of the IncA/C multidrug resistance plasmid family J Bacteriol 191 4750–4757 10.1128/JB.00189-09 . 19482926 PMC2715731

[jmm000100-Garnier1] Garnier F. Raked N. Gassama A. Denis F. Ploy M.-C. (2006). Genetic environment of quinolone resistance gene *qnrB2* in a complex *sul1*-type integron in the newly described *Salmonella enterica* serovar Keurmassar Antimicrob Agents Chemother 50 3200–3202 10.1128/AAC.00293-06 . 16940131 PMC1563520

[jmm000100-Gestal1] Gestal A.M. Stokes H.W. Partridge S.R. Hall R.M. (2005). Recombination between the *dfrA12*-*orfF*-*aadA2* cassette array and an *aadA1* gene cassette creates a hybrid cassette, *aadA8b* Antimicrob Agents Chemother 49 4771–4774 10.1128/AAC.49.11.4771-4774.2005 . 16251327 PMC1280176

[jmm000100-Hamouda1] Hamouda A. Vali L. Alsultan A. Dancer S. Amyes S.G.B. (2008). First report of ciprofloxacin resistance among *Klebsiella pneumoniae* harbouring the *qnrA1* gene and producing SHV-5 extended-spectrum β-lactamase in Scotland J Chemother 20 753–755 10.1179/joc.2008.20.6.753 . 19129076

[jmm000100-Hata1] Hata M. Suzuki M. Matsumoto M. Takahashi M. Sato K. Ibe S. Sakae K. (2005). Cloning of a novel gene for quinolone resistance from a transferable plasmid in *Shigella flexneri* 2b Antimicrob Agents Chemother 49 801–803 10.1128/AAC.49.2.801-803.2005 . 15673773 PMC547361

[jmm000100-Hochhut1] Hochhut B. Wilde C. Balling G. Middendorf B. Dobrindt U. Brzuszkiewicz E. Gottschalk G. Carniel E. Hacker J. (2006). Role of pathogenicity island-associated integrases in the genome plasticity of uropathogenic *Escherichia coli* strain 536 Mol Microbiol 61 584–595 10.1111/j.1365-2958.2006.05255.x . 16879640

[jmm000100-Holt1] Holt K.E. Thieu Nga T.V. Thanh D.P. Vinh H. Kim D.W. Vu Tra M.P. Campbell J.I. Hoang N.V.M. Vinh N.T. other authors (2013). Tracking the establishment of local endemic populations of an emergent enteric pathogen Proc Natl Acad Sci U S A 110 17522–17527 10.1073/pnas.1308632110 . 24082120 PMC3808646

[jmm000100-Hu1] Hu F.P. Xu X.G. Zhu D.M. Wang M.G. (2008). Coexistence of *qnrB4* *qnrS1* in a clinical strain of *Klebsiella* *pneumoniae* Acta Pharmacol Sin 29 320–324 10.1111/j.1745-7254.2008.00757.x . 18298896

[jmm000100-Huang1] Huang Z. Mi Z. Wang C. (2008). A novel β-lactamase gene, LAP-2, produced by an *Enterobacter cloacae* clinical isolate in China J Hosp Infect 70 95–96 10.1016/j.jhin.2008.04.012 . 18550213

[jmm000100-Jacoby1] Jacoby G.A. Walsh K.E. Mills D.M. Walker V.J. Oh H. Robicsek A. Hooper D.C. (2006). *qnrB*, another plasmid-mediated gene for quinolone resistance Antimicrob Agents Chemother 50 1178–1182 10.1128/AAC.50.4.1178-1182.2006 . 16569827 PMC1426915

[jmm000100-Kado1] Kado C.I. Liu S.T. (1981). Rapid procedure for detection and isolation of large and small plasmids J Bacteriol 145 1365–1373 . 7009583 10.1128/jb.145.3.1365-1373.1981 PMC217141

[jmm000100-Kehrenberg1] Kehrenberg C. Friederichs S. de Jong A. Michael G.B. Schwarz S. (2006). Identification of the plasmid-borne quinolone resistance gene *qnrS* in *Salmonella* *enterica* serovar Infantis J Antimicrob Chemother 58 18–22 10.1093/jac/dkl213 . 16720566

[jmm000100-Kehrenberg12] Kehrenberg C. Hopkins K.L. Threlfall E.J. Schwarz S. (2007). Complete nucleotide sequence of a small *qnrS1*-carrying plasmid from *Salmonella enterica* subsp. *enterica* Typhimurium DT193 J Antimicrob Chemother 60 903–905 10.1093/jac/dkm283 . 17652106

[jmm000100-Kim1] Kim E.H. Aoki T. (1994). The transposon-like structure of IS26-tetracycline, and kanamycin resistance determinant derived from transferable R plasmid of fish pathogen, *Pasteurella piscicida* Microbiol Immunol 38 31–38 10.1111/j.1348-0421.1994.tb01741.x . 8052160

[jmm000100-Kobayashi1] Kobayashi I. (2001). Behavior of restriction-modification systems as selfish mobile elements and their impact on genome evolution Nucleic Acids Res 29 3742–3756 10.1093/nar/29.18.3742 . 11557807 PMC55917

[jmm000100-Lavigne1] Lavigne J-P. Marchandin H. Delmas J. Bouziges N. Lecaillon E. Cavalie L. Jean-Pierre H. Bonnet R. Sotto A. (2006). *qnrA* in CTX-M-producing *Escherichia coli* isolates from France Antimicrob Agents Chemother 50 4224–4228 10.1128/AAC.00904-06 . 16982788 PMC1693975

[jmm000100-Literak1] Literak I. Dolejska M. Janoszowska D. Hrusakova J. Meissner W. Rzyska H. Bzoma S. Cizek A. (2010). Antibiotic-resistant *Escherichia coli* bacteria, including strains with genes encoding the extended-spectrum β-lactamase and QnrS, in waterbirds on the Baltic Sea Coast of Poland Appl Environ Microbiol 76 8126–8134 10.1128/AEM.01446-10 . 20952638 PMC3008254

[jmm000100-Lodwick1] Lodwick D. Owen D. Strike P. (1990). DNA sequence analysis of the *imp* UV protection and mutation operon of the plasmid TP110: identification of a third gene Nucleic Acids Res 18 5045–5050 10.1093/nar/18.17.5045 . 2129552 PMC332119

[jmm000100-Martinez-Martinez1] Martínez-Martínez L. Pascual A. Jacoby G.A. (1998). Quinolone resistance from a transferable plasmid Lancet 351 797–799 10.1016/S0140-6736(97)07322-4 . 9519952

[jmm000100-Nga1] Nga T.V.T. Parry C.M. Le T. Lan N.P.H. Diep T.S. Campbell J.I. Hoang N.V.M. Dung T. Wain J. other authors (2012). The decline of typhoid and the rise of non-typhoid salmonellae and fungal infections in a changing HIV landscape: bloodstream infection trends over 15 years in southern Vietnam Trans R Soc Trop Med Hyg 106 26–34 10.1016/j.trstmh.2011.10.004 . 22137537

[jmm000100-Nhu1] Nhu N.T.K. Lan N.P.H. Campbell J.I. Parry C.M. Thompson C. Tuyen H.T. Hoang N.V.M. Tam P.T.T. Le V.M. other authors (2014). Emergence of carbapenem-resistant *Acinetobacter baumannii* as the major cause of ventilator-associated pneumonia in intensive care unit patients at an infectious disease hospital in southern Vietnam J Med Microbiol 63 1386–1394 10.1099/jmm.0.076646-0 . 25038137 PMC4170484

[jmm000100-Park1] Park Y.-J. Yu J.K. Kim S.-I. Lee K. Arakawa Y. (2009). Accumulation of plasmid-mediated fluoroquinolone resistance genes, *qepA* *qnrS1*, in *Enterobacter aerogenes* co-producing RmtB and class A β-lactamase LAP-1 Ann Clin Lab Sci 39 55–59 . 19201742

[jmm000100-Parkhill1] Parkhill J. Wren B.W. Thomson N.R. Titball R.W. Holden M.T. Prentice M.B. Sebaihia M. James K.D. Churcher C. other authors (2001). Genome sequence of *Yersinia pestis*, the causative agent of plague Nature 413 523–527 10.1038/35097083 . 11586360

[jmm000100-Poirel1] Poirel L. Leviandier C. Nordmann P. (2006). Prevalence and genetic analysis of plasmid-mediated quinolone resistance determinants QnrA and QnrS in Enterobacteriaceae isolates from a French university hospital Antimicrob Agents Chemother 50 3992–3997 10.1128/AAC.00597-06 . 16982792 PMC1694006

[jmm000100-Poirel12] Poirel L. Cattoir V. Soares A. Soussy C.-J. Nordmann P. (2007). Novel Ambler class A β-lactamase LAP-1 and its association with the plasmid-mediated quinolone resistance determinant QnrS1 Antimicrob Agents Chemother 51 631–637 10.1128/AAC.01082-06 . 17116662 PMC1797722

[jmm000100-Rutherford1] Rutherford K. Parkhill J. Crook J. Horsnell T. Rice P. Rajandream M.A. Barrell B. (2000). Artemis: sequence visualization and annotation Bioinformatics 16 944–945 10.1093/bioinformatics/16.10.944 . 11120685

[jmm000100-Som1] Som S. Bhagwat A.S. Friedman S. (1987). Nucleotide sequence and expression of the gene encoding the *Eco*RII modification enzyme Nucleic Acids Res 15 313–332 10.1093/nar/15.1.313 . 3029675 PMC340412

[jmm000100-Strahilevitz1] Strahilevitz J. Jacoby G.A. Hooper D.C. Robicsek A. (2009). Plasmid-mediated quinolone resistance: a multifaceted threat Clin Microbiol Rev 22 664–689 10.1128/CMR.00016-09 . 19822894 PMC2772364

[jmm000100-Sumrall1] Sumrall E.T. Gallo E.B. Aboderin A.O. Lamikanra A. Okeke I.N. (2014). Dissemination of the transmissible quinolone-resistance gene *qnrS1* by IncX plasmids in Nigeria PLoS One 9 e110279 10.1371/journal.pone.0110279 . 25340787 PMC4207749

[jmm000100-Sundin1] Sundin G.W. Bender C.L. (1996). Dissemination of the *strA*-*strB* streptomycin-resistance genes among commensal and pathogenic bacteria from humans, animals, and plants Mol Ecol 5 133–143 10.1111/j.1365-294X.1996.tb00299.x . 9147689

[jmm000100-Tran1] Tran T.H. Nguyen T.D. Nguyen T.T. Ninh T.T. Tran N.B.C. Nguyen V.M.H. Tran T.T.N. Cao T.T. Pham V.M. other authors (2010). A randomised trial evaluating the safety and immunogenicity of the novel single oral dose typhoid vaccine M01ZH09 in healthy Vietnamese children PLoS One 5 e11778 10.1371/journal.pone.0011778 . 20668668 PMC2909895

[jmm000100-Vien1] Vien T.M. Baker S. Le Thi T.P. Le Thi T.P. Cao T.T. Tran T.T.N. Nguyen V.M.H. Campbell J.I. Lam M.Y. other authors (2009). High prevalence of plasmid-mediated quinolone resistance determinants in commensal members of the Enterobacteriaceae in Ho Chi Minh City, Vietnam J Med Microbiol 58 1585–1592. 19696153 10.1099/jmm.0.010033-0 PMC2884939

[jmm000100-Vien12] Vien T.M. Abuoun M. Morrison V. Thomson N. Campbell J.I. Woodward M.J. Van Vinh Chau N. Farrar J. Schultsz C. Baker S. (2011). Differential phenotypic and genotypic characteristics of *qnrS1*-harboring plasmids carried by hospital and community commensal Enterobacteria Antimicrob Agents Chemother 55 1798–1802 10.1128/AAC.01200-10 . 21282449 PMC3067134

[jmm000100-Vien123] Vien T.M. Minh N.N.Q. Thuong T.C. Khuong H.D. Nga T.V.T. Thompson C. Campbell J.I. de Jong M. Farrar J.J. other authors (2012). The co-selection of fluoroquinolone resistance genes in the gut flora of Vietnamese children PLoS One 7 e42919 10.1371/journal.pone.0042919 . 22937000 PMC3427306

[jmm000100-Wang1] Wang M. Guo Q. Xu X. Wang X. Ye X. Wu S. Hooper D.C. Wang M. (2009). New plasmid-mediated quinolone resistance gene, *qnrC*, found in a clinical isolate of *Proteus mirabilis* Antimicrob Agents Chemother 53 1892–1897 10.1128/AAC.01400-08 . 19258263 PMC2681562

[jmm000100-Wu1] Wu J.-J. Ko W.-C. Chiou C.-S. Chen H.-M. Wang L.-R. Yan J.-J. (2008). Emergence of Qnr determinants in human *Salmonella* isolates in Taiwan J Antimicrob Chemother 62 1269–1272 10.1093/jac/dkn426 . 18957397

[jmm000100-Zhao1] Zhao F. Bai J. Wu J. Liu J. Zhou M. Xia S. Wang S. Yao X. Yi H. other authors (2010). Sequencing and genetic variation of multidrug resistance plasmids in *Klebsiella pneumoniae* PLoS One 5 e10141 10.1371/journal.pone.0010141 . 20405037 PMC2853573

